# A Comprehensive Investigation of the Mechanical and Tribological Properties of AZO Transparent Conducting Oxide Thin Films Deposited by Medium Frequency Magnetron Sputtering

**DOI:** 10.3390/ma17010081

**Published:** 2023-12-23

**Authors:** Michał Mazur, Milena Kiliszkiewicz, Witold Posadowski, Jarosław Domaradzki, Aleksandra Małachowska, Paweł Sokołowski

**Affiliations:** 1 Faculty of Electronics, Photonics and Microsystems, Wrocław University of Science and Technology, Janiszewskiego 11/17, 50-372 Wroclaw, Poland; michal.mazur@pwr.edu.pl (M.M.); milena.kiliszkiewicz@pwr.edu.pl (M.K.); witold.posadowski@pwr.edu.pl (W.P.); 2 Faculty of Mechanical Engineering, Wrocław University of Science and Technology, Lukasiewicza 5, 50-371 Wroclaw, Poland; aleksandra.malachowska@pwr.edu.pl (A.M.); pawel.sokolowski@pwr.edu.pl (P.S.)

**Keywords:** AZO, transparent conducting oxide, thin film, magnetron sputtering, hardness, nanoindentation, scratch resistance, optical properties, resistivity, figure of merit

## Abstract

This paper presents a detailed analysis of aluminium-doped zinc oxide (AZO) thin films and considers them a promising alternative to indium tin oxide in transparent electrodes. The study focusses on critical properties of AZO, including optical, electrical, and mechanical properties, with potential applications in displays, photovoltaic cells, and protective coatings. The deposited AZO thin films are characterised by excellent optical and electrical parameters, with transparency in the visible light range exceeding 80% and resistivity of 10^−3^ Ω·cm, which gives a high value of figure of merit of 63. Structural analysis confirms the nanocrystalline nature of as-deposited AZO thin films, featuring hexagonal ZnO, orthorhombic Al_2_O_3_, and cubic Al_2_ZnO_4_ phases. The study includes nanoindentation measurements, which reveal exceptional hardness (11.4 GPa) and reduced elastic modulus (98 GPa), exceeding typical values reported in the literature, highlighting their protective potential. Abrasion tests have shown extraordinary scratch resistance due to the lack of impact on topography and surface roughness up to 10,000 cycles. This comprehensive study demonstrated that as-deposited AZO thin films are multifunctional materials with exceptional optical, electrical, and mechanical properties. The findings open up possibilities for a variety of applications, especially in protective coatings, where the combination of hardness, scratch resistance, and transparency is both rare and valuable.

## 1. Introduction

Aluminium-doped zinc oxide (AZO) thin films constitute an excellent alternative to indium tin oxide (ITO), widely used today as a transparent electrode (TCO) in various types of devices. The primary range of applications for such TCO electrodes is in various types of displays (LCD, OLED), thin-film photovoltaic cells, heating layers, transparent shielding layers, etc. [1,2,3,4]. The main requirements that materials for such electrodes should meet are excellent transparency and electrical conductivity.

AZO is an example of a TCO material that is characterised by very good optical and electrical performance [5,6] with a generally higher transparency of over 80% and a resistivity in the range of 10^−4^ cm [7,8]. For example, Agura et al. [9], obtained even a resistivity of as low as 8.54 × 10^−5^ Ω∙cm and a transmittance of 88% in the visible spectrum for AZO thin films manufactured using laser-pulsed deposition.

The evaluation of the mechanical properties of such thin films is another extremely important task. Additional requirements that are commonly met in practice include, among others, good adhesion to the substrate and good mechanical (tribological) properties, which determine the applicability of such a TCO material, for example, simultaneously as a protective layer. Hence, thin films of such materials should also be characterised by good abrasion resistance and adequate hardness. The mechanical parameters of the films are mainly influenced by the surface morphology, the level of doping, and the crystal structure [10]. Huang and Chang [11] noted that the mechanical properties of thin films are also strongly affected by the type of substrate used.

For manufacturing of AZO thin films, various techniques are used. However, magnetron sputtering seems to be the most commonly used [5,12,13]. The sputtering offers manufacturing of homogeneous thin films with high repeatability, excellent performance, and a high deposition rate over a large area (even hundreds of metres square) that is not achievable using other techniques. In addition to magnetron sputtering, chemical vapour deposition (CVD) [14], atomic layer deposition (ALD) [15], spin coating [16], the sol-gel method [17] or pulsed laser deposition [18] are also often mentioned.

Pat et al. [19] noted that doping ZnO with aluminium improves the hardness of the films. In the study, the hardness of the AZO films manufactured using the RF sputtering deposition technique was determined from 7 to 11 GPa. Young’s modulus was 155 and 95 GPa, respectively, indicating that this parameter decreases with ZnO doping of aluminium. Tests on AZO films [20] manufactured by radio frequency magnetron sputtering and further annealed in the range from 300 °C to 500 °C increased their hardness from 7.1 GPa to 11.2 GPa while Young’s modulus was 98.6 GPa and 122 GPa [20]. The study indicated that at higher annealing temperatures, the grain size was larger and the average surface roughness increased from 7.6 nm to 9.5 nm [20]. Chang et al. [21] noted that at low sputtering power and substrate temperature, the AZO films obtained by magnetron sputtering were characterised by a spherical grain structure. As the power and substrate temperature increased, the grains changed in the orientation of the crystallites. Nanoindentation studies indicated an increase in hardness from 8 to 10 GPa with a higher sputtering power. Chang et al. believe that this is due to the higher density and crystallinity of the resulting structures [21]. The hardness of the AZO films was also studied by Lin et al. [22]. They used films manufactured by RF magnetron sputtering on glass substrates. The substrate temperature was varied during the process. They used room temperature, 150 °C, and 300 °C, and the hardness of the films was 7.06 GPa (for room temperature), 5.08 GPa (for 150 °C), and 4.78 GPa (for 300 °C). The test performed indicated that increasing the substrate temperature during deposition lowered the hardness of the thin film thus obtained. The elastic modulus was 92.15, 87.9 and 91.21 GPa, respectively. Similarly, the results presented in [23] for AZO thin films deposited on glass substrates using a reactive midfrequency (MF) magnetron sputtering process showed that as the substrate temperature increases (from 50 °C to 300 °C), the hardness of the AZO films also increases (from 7.3 GPa to 9.5 GPa). Hong et al. [23] believe that this is due to the better crystallinity of the AZO films, which improves as the substrate temperature increases. Wen et al. [24] also studied the effect of substrate temperature during deposition on the hardness of AZO films. They noted that the lower the temperature, the higher the hardness. The AZO films were manufactured by RF magnetron sputtering, and the hardness was 5.6 GPa for room temperature, 4.08 GPa for 200 °C, 4.46 GPa for 300 °C, and 4.22 GPa for 400 °C. Kuriki and Kawashima [20] manufactured AZO films by DC magnetron sputtering. They obtained a hardness of 6.0 GPa to 6.7 GPa. AZO films manufactured at different working pressures (from 1 mTorr to 15 mTorr) by pulsed DC sputtering were studied by Kar et al. [25]. They used a cylindrical AZO target (2 wt. %) and argon (5N) as a sputtering gas. Mechanical tests revealed that the highest hardness occurs for 3 mTorr [25]. The hardness of the AZO films was also analysed by Koidis et al. [26], who manufactured the films using DC pulsed. The AZO hardness obtained was in the range from 6.5 to 9.1 GPa depending on different sputtering power (135 W, 200 W, 300 W). They noticed that the higher the power, the higher the hardness. Zhu et al. [27], manufactured AZO films by DC magnetron sputtering. They used a high purity (99.999%) of the AZO target (2% wt. Al_2_O_3_) and a substrate temperature of 280 °C. The pressure was 1.1 Pa and the argon flow rate was 15 sccm. The hardness of the films was 10.2 GPa [27], which is one of the highest observed for films of this type.

In case of the ALD technique, AZO thin films manufactured at low temperature showed a hardness of 8.22 GPa [28]. Lai et al. [29] studied the mechanical properties of AZO films manufactured also by the sol-gel method. They subjected the obtained films to rapid thermal annealing at temperatures of 350 °C, 400 °C, and 600 °C. The hardness of the films was observed to increase with increasing thermal annealing temperature from 4.02 GPa (for 350 °C) to 4.81 GPa (for 600 °C).

For AZO manufactured by plasma-enhanced CVD at atmospheric pressure, a hardness of 3.7 GPa was achieved [30]. AZO films were also manufactured using a solution of zinc acetate dehydrate doped with aluminium chloride by spin coating. The films were deposited at 3000 RPM for 30 s and annealed at 500 °C for 10 min. As the grain size increased as a result of annealing, the hardness decreased. For example, a hardness of 9.3 GPa was obtained for a grain size of 230 Å [31].

The short literature review showed that the mechanical properties of AZO thin films are strongly related to many different factors, including the manufacturing technology and the parameters used during deposition. However, the hardness varies, on average, from 4 GPa (low) for thin films prepared using CVD and sol gel through 5–7 GPa (medium) for sputtered on heated substrates to 8–11 (relatively high) for sputtered on unheated substrates. The hardness of the AZO films depending on the method of manufacture and the parameters used during the process discussed in the present review are collected in Table 1.

Investigations on mechanical performance include also reliability analysis on the percentage change in resistance and the appearance of cracks. Hamasha et al. [33], analysed the manufacturing of AZO films by RF magnetron sputtering (75 W/5 min) with a thickness of 425 nm on Kapton polyimide substrates (125 µm thick). In the process, they used a high purity (99.99%) target (99.99%) with a composition of 98% wt. ZnO and 2% wt. Al_2_O_3_. Films deposited on elastic Kapton foils were subjected to cyclic bending. They studied the change in resistance after 50, 100, 200, 300, 400 and 500 bending cycles. They noted that the percentage change in electrical resistance is greater at lower bending radiuses. The percentage change in electrical resistance increases as the number of cycles increases. The study also indicated that cracks formed more in the centre of the structures studied than on their sides [33].

The growing interest in the thin film coating industry results from the emergence of new applications and changing customer requirements. In addition to the primary requirement for TCO films, that is the transparency, there is a need for TCO coatings that can simultaneously integrate additional functionalities. This has resulted in a shift towards creating coatings that not only serve as a transparent electrode, but also offer enhanced functions to meet a variety of requirements, for example, increased hardness. In the case of the present work, the AZO thin films prepared using medium frequency magnetron sputtering were subjected not only to optical and electrical tests but also to check their hardness and scratch-resistance as a perspective for use as a protective coating. To the best of our knowledge, such hard, scratch-resistant, and transparent conducting features are very rarely seen or described.

## 2. Materials and Methods

A NA500 PVD system (Boleslawiec, Poland) with a typical 500 mm diameter, 600 mm high bell-jar-type working chamber was used for thin-film deposition. The self-made disc-type magnetron based on NdFeB magnets (internal N pole and external S pole) that ensures the operation in the balanced mode was mounted at the base of the vacuum chamber. Our previous work [5] presented the results of studies on the electrical properties, optical properties, chemical composition, and microstructure of AZO thin films prepared using a self-made two-element Zn-Al target. The effect of the position of the substrate in relation to the target and the sputtering conditions was investigated. The results showed that favourable conditions for the formation of TCO layers required placing the substrates at a distance of more than 5 cm from the axis of the target (in a plane 9 cm away from the surface of the target)—the so-called off-axis mode. In contrast to the studies presented in the article [5], in the present study a commercial high purity target (from ITL Vacuum, 107 mm in diameter and 6 mm thick) with a composition of 99.9%, consisting of 98% by weight ZnO and 2% by weight Al_2_O_3_ was used in the present study. The substrates were placed 9 cm in a plane parallel to the target surface and approximately 7.5 cm from the target axis (off-axis mode). The energy necessary for the sputtering was provided by a pulsed Dora Power Systems (Wroclaw, Poland) power supply (MSS-10kW model) operating at a frequency of 100 kHz. Film deposition was performed using a vacuum chamber equipped with a diffusion pump with a high pumping speed of 2000 L/s, complemented by a rotary pump with a volumetric pumping capacity of 30 m^3^/h. It should be noted especially that the authors designed a magnetron with a conventional circular configuration, using NdFeB magnets characterised by an inner pole N and an outer pole S. Such a magnetic arrangement ensured balanced and uniform operation during the sputtering process, contributing to the precision and reliability of the film deposition procedure. The deposition process was carried out using argon. Initially, the working chamber was evacuated to a background pressure of 1.6 × 10^−3^ Pa. Subsequently, argon was introduced, adjusting the internal chamber pressure to 2.6 × 10^−1^ Pa. The power supplied to the target during discharge was 100 W and was augmented by a pulsed medium-frequency power supply, inducing a pulsed magnetron discharge. The power supply generated sinusoidal output current pulses with a duration of 10 µs and a stabilised amplitude of 16 A. To preserve the sinusoidal profile of the magnetron current throughout each pulse, the power supply’s output voltage was dynamically adapted in response to fluctuations in the impedance of the magnetron discharge. The deposition process takes 35 min. The deposition parameters used in the process were selected based on our previous experience [5] and are succinctly described in Table 2.

The thickness of the thin film deposited, measured using the Tylor Hobson CCI Lite optical profiler (Tylor Hobson, Leicester, UK) and a step height method, was 380 nm ± 5 nm. The weight concentration of Al in the prepared AZO thin films evaluated using the energy dispersive spectrometer attached to the scanning electron microscope was equal to 3.2 wt. %.

Structural studies were performed using the Empyrean PIXel3D diffractometer (Panalytical, Malvern, UK). X-ray diffraction (XRD) patterns were acquired in the grazing incidence mode (GIXRD) at an angle of 3 degrees with Cu Kα radiation (wavelength of 0.15406 nm). The obtained diffraction patterns were subjected to analysis using MDI JADE 5.0 software, allowing for a complete interpretation and characterisation of the structural properties of the examined samples. The surface and cross-sectional morphology of the AZO thin films was investigated using a scanning electron microscope (FEI Helios NanoLab 600i, Lausanne, Switzerland). SEM observations were made using the so-called ‘through the lens detector’ with an acceleration voltage of 2 kV, a current of 0.17 nA and a working distance of 4.7 mm. The topography and surface roughness of the thin films was examined utilising an atomic force microscope (AFM), specifically the Nanosurf FlexAFM (Liestal, Switzerland). The analysis of the acquired data was performed using the WSxM 5.0 Develop 10.2 software package, which facilitates the comprehensive evaluation and interpretation of the surface features of the thin films. The surface resistance of the deposited thin films was determined using a standard four-point measuring head (Jandel Engineering Ltd., Leighton Buzzard, UK) and a source measurement unit (Keithley 2611A type, Keithley Instruments Inc., Cleveland, OH, USA). The measuring head was equipped with four tungsten carbide needles, which were arranged in a line with an interneedle distance of 1.00 mm. The optical properties were evaluated on the basis of transmission spectra. Characteristics were obtained using OceanOptics spectrophotometers (type QE65000 and NIR256-2.1) (Ocean Optics, Dunedin, FL, USA) and a coupled halogen-deuterium lamp (DH-2000-BAL) (Ocean Optics, Dunedin, FL, USA). Transmission was measured in the range of 300–1800 nm. The transmitted light was collected using a directional array. The optical measurement was performed before the mechanical tests of the samples and after the abrasion tests. The mechanical properties of the AZO films were systematically characterised using a nanoindentation approach facilitated by an Anton Paar Tester NHT3 nanoindenter mounted on the STeP 4 platform, employing a Berkovich type indenter (Anton Paar GmbH, Graz, Austria). The experimental results were subjected to analysis using the methodology advanced by Oliver and Pharr [34,35,36]. The micromechanical characteristics of each thin film were assessed by conventional load-controlled tests and continuous stiffening measurements (CSM), a significant improvement in the nanoindentation methodology [34,35,37,38,39,40]. In the examination of the mechanical attributes of thin films utilising nanoindentation, careful consideration was given to the potential influence of the substrate on the obtained results. The widely adopted “10% principle” [41] was used to mitigate substrate effects, stipulating that the nanoindentation depth should not exceed 10% of the thickness of the measured films. The application of the “10% principle” encounters impracticalities in the context of thin films, frequently characterised by thicknesses below 600 nm. Adherence to this principle for such films would necessitate nanoindentation depths below 60 nm, a range that is susceptible to significant errors due to prevailing technological limitations. Consequently, alternative methodologies are imperative to address these challenges.

The scientific literature extensively discusses several mathematical models tailored for the comprehensive performance and analysis of nanoindentation values in thin films [42,43,44,45]. The measured hardness of thin films deposited on substrates can be systematically expressed as a power-law function, where substrate hardness, thin film hardness, nanoindentation depth, and thin film thickness are integral variables, as encapsulated by Equation (1) [45].

(1) $$H=H_{s}\cdot {\left(\frac{H_{F}}{H_{s}}\right)}^{\frac{1}{1+A{\left(\frac{h}{d}\right)}^{B}}}$$

where: *H_F_*—hardness of thin films; *H_s_*—hardness of substrate; *A*, *B*—adjustable coefficients, *h*—maximum indenter displacement, *d*—thickness of thin film.

Equation (1) is required to adhere to pivotal boundary conditions: as the indentation depth asymptotically approaches zero, indicative of the diminutive indentation displacements, the measured hardness is expected to approach the intrinsic hardness of the thin film. On the contrary, when the indentation depth converges toward the thickness of the thin film, the measured hardness is anticipated to approach the inherent hardness value of the substrate. These prescribed boundary conditions assume significance in the precise interpretation of results in nanoindentation studies, thereby furnishing indispensable insights into the mechanical characteristics of thin films.

The TABER^®^ oscillating abrasion tester Model 6160 (North Tonawanda, NY, USA), was used to evaluate the abrasion and scratch resistance of the materials. The analysed sample was attached to the bottom of a moving tray containing silica sand. The platform performed reciprocating motions of 100 mm in length, at a speed of 150 cycles per minute. The roughness of the films was analysed and microscopic images were taken after 600, 1200, 2400, 4800, 9600, 19,200, 38,400 and 57,600 cycles. Microscopic analysis was performed using an Olympus BX51 microscope at a magnification of 10×. A Taylor-Hobson TalySurf CCI Lite profilometer (Taylor Hobson, Leicester, UK) was used for roughness analysis.

## 3. Results

### 3.1. Optical and Electrical Properties

**Figure 1:**
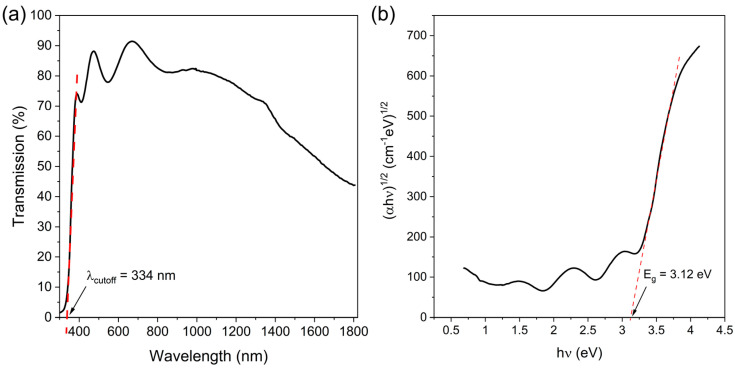
Transmission characteristic (**a**) and Tauc plot for indirect allowed transitions (**b**) of prepared AZO thin film with indicated position of fundamental absorption edge (λ_cutoff_) and optical band gap width (E_g_).

Figure 1a presents the optical transmission spectra of the as-prepared thin film. It can be seen that the prepared thin films had excellent transparency in the visible wavelength range of optical radiation above λ_cutoff_ = 334 nm. The average transparency in the visible range (from 360 to 760 nm) is 82.4%. Based on the measured transmission characteristic, the width of the optical energy gap for the allowed indirect transitions was estimated using the Tauc approximation (Figure 1b), which in this case is 3.12 eV. The position of the optical edge is very sharp, indicating a very good structural quality of the prepared films. On the basis of the slope of the absorption edge, the so-called Urbach energy (E_u_) was estimated as well. The smaller the value of this energy, the smaller the width of the tails of the extended states in the forbidden gap. In the case under study, E_u_ was estimated at about 0.16 eV, which is a relatively small value demonstrating good microstructure quality.

In Figure 1a, a decrease in transparency in the wavelength range above 1000 nm is visible, which is a characteristic feature of conductive oxides. This effect is due to the absorption of photons by free electrons (Drude absorption), and is an indicator of the good electrical conductivity of the manufactured films. The resistivity of the fabricated AZO film was 2.6 × 10^−3^ Ω∙cm, a value comparable to those reported in the literature (e.g., [46]). One of the parameters indicating the good properties of the material as a transparent oxide conductor is the so-called FOM (figure of merit), which takes into account both transparency and conductivity. For the purpose of this work, to make the results independent of the thickness of the prepared film, we used the formula for FOM in the following form:

(2) $$FOM=\frac{T^{10}}{\rho }$$

where: *T*—transmission (from 0 to 1), and *ρ*—resistivity in Ω∙cm.

For the manufactured film, the FOM was equal to 63, which makes it a good TCO material [47]. The key parameter to obtain AZO thin films as a good TCO material is the power supplied to the magnetron during the sputtering process. As a result of our own experience, in order to obtain transparent films with the highest electrical conductivity, the power released in the target should not exceed just several W/cm^2^ (<3 W/cm^2^). Too much power generally resulted in less conductive films. The results of the optical and electrical properties are summarised in Table 3.

### 3.2. Microstructure Properties

**Figure 2:**
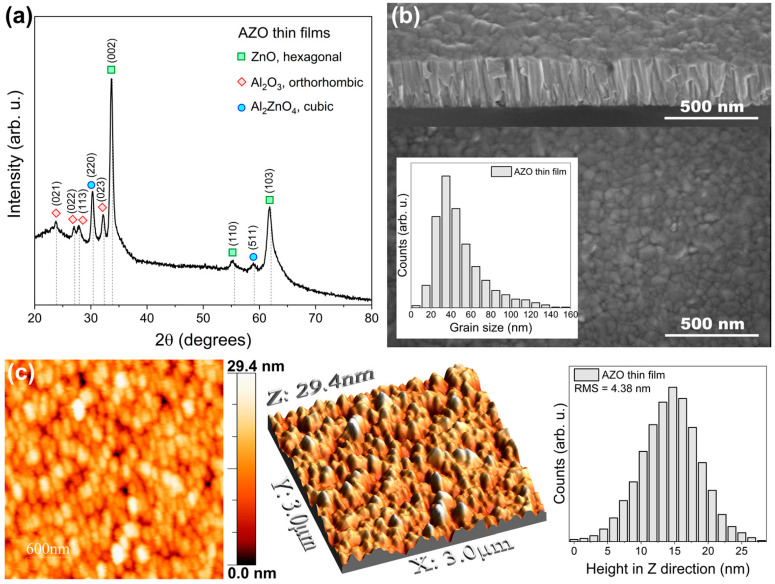
Investigation results of magnetron sputtered AZO thin films: (**a**) XRD pattern, (**b**) SEM images of surface and cross-section morphology, (**c**) AFM images of surface and height distribution in Z direction.

Figure 2a shows the GIXRD pattern obtained for the AZO thin film deposited on a fused silica substrate. Three different crystalline phases can be seen in the pattern, namely hexagonal ZnO, orthorhombic Al_2_O_3_ and cubic Al_2_ZnO_4_. The observed peaks are very well visible, sharp and of rather high intensity, indicative of the robust crystalline nature of the thin film. This observation aligns with the findings from Urbach energy analysis. Using the Scherrer formula [48], the crystallite size analysis demonstrated the nanocrystalline nature of the AZO thin film. Specifically, the crystallite sizes corresponding to ZnO (calculated for the (002) plane), Al_2_O_3_ (calculated for the (023) plane), and Al_2_ZnO_4_ (calculated for the (220) plane) were determined to be approximately 14.0 nm, 17.7 nm, and 16.2 nm, respectively. The surface and cross-sectional morphology of the AZO thin film was analysed using FE-SEM, as shown in Figure 2b. Notably, the analysis revealed a smooth surface characterised by uniformly distributed nanograins of quite uniform size. The distribution of the grain size is shown as an inset and the authors testify that most of the grain size is in the range from 30 to 50 nm. Cross-sectional examination further revealed a very dense, columnar growth of an AZO thin film. AFM was employed for topographical assessment of the thin films. Figure 2c presents two- and three-dimensional images of the surface, including the height distribution of grains. Interestingly, the surface was crack-free and the grains exhibited a maximum height of approximately 30 nm. AFM images confirmed the uniform distribution of grains on the surface of the thin film, consistent with the SEM findings. Furthermore, using height distribution data, the surface roughness of the root mean square (RMS) was calculated and determined to be 4.38 nm.

**Figure 3:**
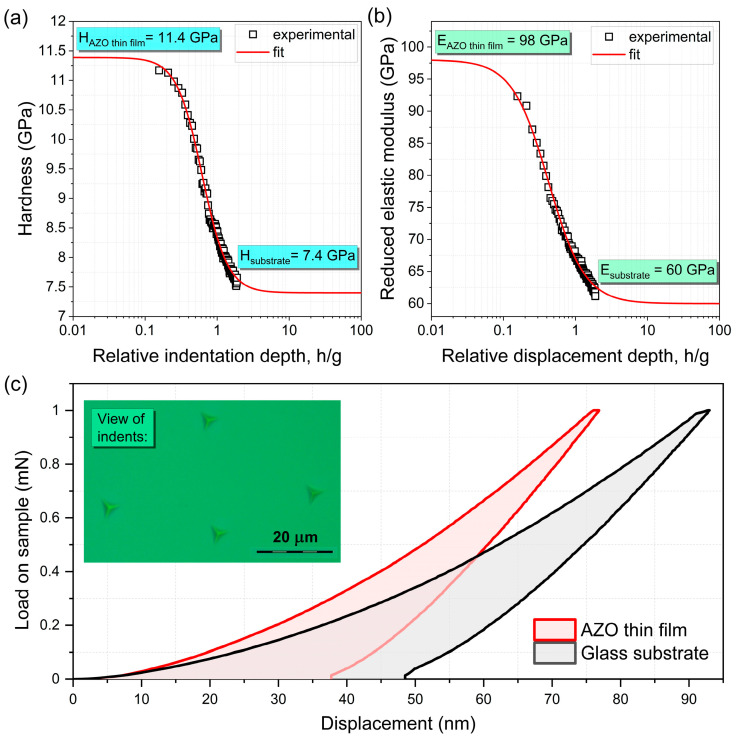
Investigation results of mechanical studies: (**a**) hardness, (**b**) reduced elastic modulus and (**c**) comparison of traditional load-displacement curves of AZO thin film and glass substrate.

In the case of CSM nanoindentation testing, five measurements were conducted at various places on the thin film. Consistent results from these measurements may suggest the excellent homogeneity of the as-deposited AZO thin films. Figure 3a,b show the hardness and reduced elastic modulus, respectively, with values of 11.4 GPa and 98 GPa. These values surpass those of the Corning glass substrate by more than 50% in both cases, indicating the formidable protective properties that the AZO thin film can offer to glass substrates. This is particularly noteworthy when considering also metallic materials, such as the biomedical TiAlV alloy, whose hardness is less than 5 GPa.

In addition, traditional nanoindentation experiments were performed, including 20 measurements on both the AZO thin film and the Corning substrate, each with a maximum load of 1 mN. Figure 3c shows a comparison of traditional load-displacement curves for AZO thin film and a glass substrate, along with exemplary images of the indents (inserted in the graph). It can be concluded that under the same loads, the displacement of the indenter tip into the material is smaller in the case of Corning covered with an AZO thin film than without it. This testifies to the higher hardness of the thin film compared to that of the substrate. Moreover, the results of the hardness and elastic modulus are consistent with the CSM technique, with only negligible differences between the traditional and CSM measurements.

The purpose of this paper is to provide a comprehensive characterisation of thin films consisting of aluminium-doped zinc oxide (AZO), with particular emphasis on their hardness and elastic modulus properties. An extensive review of the literature [19,20,21,22,23,24,26,27,28,29,30,31,32] indicates that typical hardness values of AZO layers range from 10 GPa, depending on the technology used. A detailed comparison of these values is presented in Table 1. The results of the experimental test showed that the AZO films achieved a remarkable hardness of 11.4 GPa, exceeding the established reference values. This finding highlights the extraordinary mechanical efficiency inherent in the studied AZO films, which potentially reveals new possibilities for their applications. We believe that there are several factors that might influence the observed high hardness value. They are, but not limited to, nanocrystalline behaviour of deposited films, and futures of the used magnetron sputtering process itself. As stated by the results obtained from XRD measurements, the nanocrystalline structure was composed from crystallites of three different crystal phases, i.e., ZnO, Al_2_O_3_ and Al_2_ZnO_4_. The crystallite size of these phases is in the range from ca. 14 to 18 nm and the high intensity of the peaks seen in the XRD pattern testify to the good crystalline quality of the thin films. The hardness can be increased by depositing thin films with a dense structure, while for nanomaterials it is possible to achieve a hardness even greater compared to the bulk ones [49,50]. This is because the hardness of the material increases when the grain size or the crystallite size decreases with the maximum in the nanometre range (Hall-Petch effect) [51]. In our case, with such dimensions of crystallites in the range of several nanometres, the Hall-Petch effect might be one of the factors increasing the hardness of AZO. Furthermore, the hardness of Al_2_O_3_ thin films can reach 15–20 GPa [52,53]. Therefore, the presence of Al_2_O_3_ phase in our AZO coatings may influence and increase their overall hardness. Similarly, as the grain size decreases in nanocrystalline materials, the hardness generally increases. This is because smaller grains hinder the movement of dislocation, leading to improved mechanical properties. Additionally, the presence of grain boundaries may be crucial because a higher density of grain boundaries can impede the motion of the dislocation, contributing to increased hardness. In the case of our thin films, the structure is highly nanocrystalline and due to the cross-sectional SEM images (Figure 2b) it is clearly seen that AZO had a very densely packed columnar morphology, which could increase the hardness. Finally, the methods used for deposition and fabrication of nanocrystalline thin films, such as physical vapour deposition, i.e., magnetron sputtering, can impact the final hardness. In our previous studies on the properties of ZnO thin films [54], it was shown that using the magnetron sputtering process we were able to deposit hard and scratch resistant coatings with extraordinary adhesion to the substrate. In the case of undoped zinc oxide, the hardness obtained in the best thin films was equal to 11.5 GPa.

Moreover, the results of the elastic modulus analysis are particularly remarkable. Existing literature reports an elastic modulus range of 95 to 155 GPa for AZO films [19,20], while the films obtained and examined in this study showed an impressive elastic modulus value of 98 GPa. This proves that this material, in addition to its exceptional hardness, is characterised by exceptionally high elasticity. These distinctive properties become critical in semiconductor technologies, where the mechanical strength and flexibility of the materials are key determinants.

**Figure 4:**
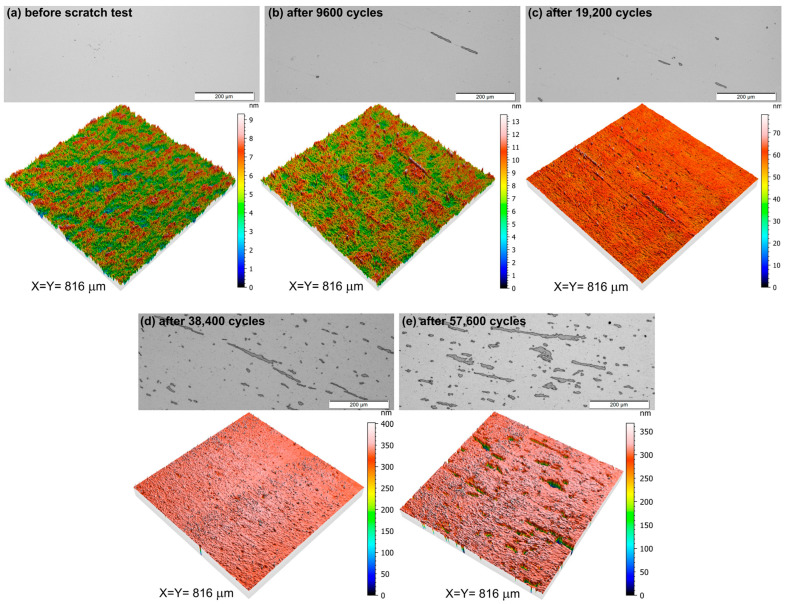
Images of the surface of AZO thin film obtained using an optical microscope and profilometer (**a**) before the scratch test and after various numbers of scratch test cycles: (**b**) 9600, (**c**) 19,200, (**d**) 38,400 and (**e**) 57,600.

Furthermore, the abrasion resistance of each deposited thin film was estimated on the basis of the Bayer test. This parameter is particularly important in the case of protective transparent coatings that may be applied in various fields of optics or optoelectronics, e.g., in ophthalmics or touch screens. The investigation results, by means of surface images, are shown in Figure 4. Measurements were acquired with the use of an optical microscope working in reflection mode and a profilometer to obtain the 3D images along with changes in surface topography. The AZO thin film was measured before and after the scratch test with an increasing number of test cycles, starting from 600 cycles and ending with 57,600 cycles. The AZO thin film had a uniform and crack-free surface before the scratch test with a root mean square roughness (S_q_) of 1.07 nm. Measurements performed after 600, 1200, 2400, 4800 test cycles showed no significant changes in the surface roughness, i.e., S_q_ maintained below 2 nm, and no scratches were found on the thin film. Increasing the number of test cycles to 9600 caused the appearance of the first few scratches of quite a small depth, however, this did not have a significant impact on the transparency or S_q_, which indicates the extraordinary scratch resistance of the AZO thin film. In turn, doubling the number of cycles ultimately resulted in an increase in the number of scratches with a simultaneous slight increase in S_q_ to 3 nm. Further increase in test cycles to 38,400 and 57,600 caused further deterioration of the surface, increasing the number of severe scratches with a depth equal to the thickness of the AZO thin film, which means that the coating was scratched from the substrate.

**Figure 5:**
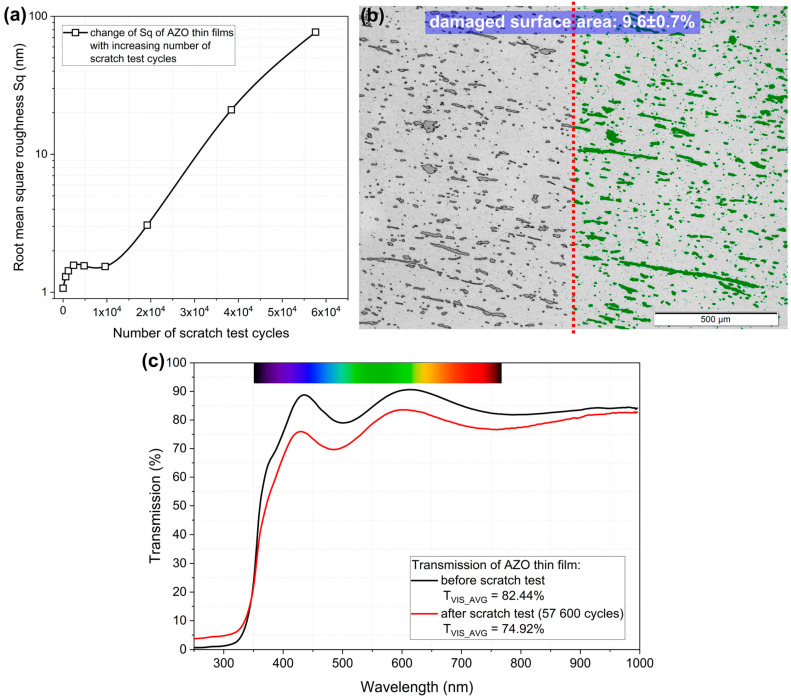
Analysis results of the AZO thin film properties after scratch test: (**a**) change in the Sq parameter with increasing number of test cycles, (**b**) image from an optical microscope with marked areas of the damaged surface after 57,600 test cycles and (**c**) change in the transmission spectrum after 57,600 test cycles.

Based on data obtained using an optical profilometer, the roughness of the root mean square surface was determined, which is presented in Figure 5a as a function of the number of test cycles. It can be seen that up to approximately 10,000 cycles, S_q_ remained stable and only increased slightly after 20,000 cycles. The AZO coating began to scratch only after the test cycles were doubling to several tens of thousands of test cycles, which is a very good result demonstrating very good adhesion to the substrate and abrasion resistance. Furthermore, using an optical microscope and Olympus Stream Essentials software ver. 1.7 (Olympus Soft Imaging Solutions GMBH, Münster, Germany), it was possible to assess that after 57,600 test cycles, the damaged surface represented approximately 9.6% of the total coating surface (shown in Figure 5b as green filling of the scratch areas). In the case of an AZO thin film treated as a transparent conductive oxide, the most important parameters are the average transparency in the visible wavelength range and the resistivity at room temperature. As-deposited thin films showed very good results in both parameters and were also measured for the thin film after 57,600 abrasion test cycles. It is worth noting that the thin film was still transparent, although it contained deep scratches. The decrease in transparency level was approximately 8% (Figure 5c). It is also worth noting that in the UV wavelength range there is a slight increase in transmission, which may be the result of scratches with a depth of AZO thickness. This means that the fused silica substrate was exposed to deep scratches of AZO, thus allowing ultraviolet (UV) light to pass through the sample. Moreover, the resistivity of the thin films measured after abrasion tests showed an increase of slightly more than twofold. This, in turn, caused the figure of merit to decrease to 11.9, which is still a very good value. Such results clearly demonstrate that the AZO thin film can still be used as a transparent conductive oxide having high transparency and low resistivity.

The summary of the investigation results of the structural, optical, electrical, and mechanical properties of AZO thin films deposited by medium frequency magnetron sputtering is presented in Table 3.

## 4. Conclusions

This paper focusses on the comprehensive characterisation of aluminium-doped zinc oxide (AZO) thin films, highlighting their optical, electrical, and mechanical properties. The study uses a circular magnetron, using a high-purity target composed of 98% ZnO and 2% Al_2_O_3_. The novelty of the presented research consists of obtaining in the AZO thin films as deposited excellent TCO properties (average transmittance in VIS 81.4%, resistivity 2.6 × 10^−3^ Ω∙cm and FOM = 63) and the impressive hardness (11.4 GPa) and reduced elastic modulus (98 GPa), exceeding typical values reported in the literature. The study further evaluated the abrasion resistance of AZO films and revealed their extraordinary scratch resistance. Thin films maintain very similar surface roughness stability for up to approximately 10,000 cycles, demonstrating their good adhesion to the substrate. It was suggested that the observed enhanced mechanical properties are due to the presence of hexagonal ZnO, orthorhombic Al_2_O_3_ and cubic Al_2_ZnO_4_ phases in the films, and to their nanocrystalline nature due to crystallite sizes in the range from ca. 14 to 18 nm.

Despite being subjected to extensive abrasion testing, the films maintain high transparency with only a slight decrease of approximately 8%. The resistivity increases twofold, but the figure of merit remains at a level of 11.9, which is still a very good value.

In conclusion, the study presents AZO thin films deposited by medium-frequency magnetron sputtering as a promising material with exceptional optical, electrical, and mechanical properties. The findings suggest potential applications in areas requiring transparent and mechanically durable coatings, opening new opportunities for AZO films in various technological areas. The conclusions drawn from this research article indicate that AZO films hold promise for advanced materials science applications, offering potential opportunities to produce highly efficient electronic devices and also for application on elastic substrates and photovoltaic components.

## Figures and Tables

**Table 1:** The hardness of the AZO films depending on the manufacturing method.

Manufacturing Method	Hardness (GPa)	References
RF Sputtering	8–10	[21]
	7–11	[19]
	7	[20]
	7.06 (for room temperature), 5.08 (for 150 °C), and 4.78 (for 300 °C)	[22]
	5.6–4.22 (from room temperature to 400 °C)	[24]
DC Sputtering	10.2	[27]
	6.0–6.4	[32]
	6.0–6.8	[26]
	7.3–9.5	[23]
Atomic Layer Deposition	8.22	[28]
Sol-Gel Method	4.02 (for 350 °C); 4.07 (for 400 °C); 4.81 (for 600 °C)	[29]
CVD	3.7	[30]
Spin Coating	9.3	[31]

**Table 2:** Summary of the deposition process parameters.

Base Pressure (Pa)	1.6 × 10^−3^
Working pressure (Pa)	2.6 × 10^−1^
Magnetron discharge power (W)	100
Deposition time (min)	35
Target diameter (mm)	107
Target thickness (mm)	6
Target purity (%)	99.9
Target composition	ZnO/Al_2_O_3_ (98/2 wt. %)
Target to substrate distance (cm)	9
Substrate temperature	Unheated—nearly room temperature

**Table 3:** Summary of the investigation results of AZO thin film.

Properties	Parameter		Result
Structural	Crystallite size (nm)		ZnO (002) = 14.0 ± 0.9 Al_2_O_3_ (023) = 17.7 ± 1.9 Al_2_ZnO_4_ (220) = 16.2 ± 1.2
Optical	Average transmittance in VIS (%)		82.4 ± 0.5
	Band gap energy, indirect transitions (eV)		3.12 ± 0.05
	Cut-off wavelength (nm)		334 ± 2
	Urbach energy (eV)		0.17 ± 0.05
Electrical	Sheet resistance (Ω/□)		68 ± 2
	Resistivity (Ω·cm)		2.6 ± 0.1 × 10^−3^
	Figure of merit		63.0
Mechanical	Hardness (GPa)		11.4 ± 0.4
	Young’s elastic modulus (GPa)		98.0 ± 4.1
	Abrasion resistance	S_q_ before scratch test (nm)	1.1 ± 0.1
		S_q_ after scratch test (nm)	76.8 ± 3.9
		Resistivity after scratch test (Ω∙cm)	4.7 × 10^−3^
		Average transmittance in VIS after scratch test (%)	74.92 ± 0.5
		Figure of merit after scratch test	11.9

## Data Availability

Data underlying the results presented in this paper are not publicly available at this time but may be obtained from the authors upon reasonable request.
